# GC/MS and PCA Analysis of Volatile Compounds Profile in Various *Ilex* Species

**DOI:** 10.3390/molecules30214230

**Published:** 2025-10-29

**Authors:** Anna Zwyrzykowska-Wodzińska, Piotr Okińczyc, Jakub Szperlik, Bogdan Jarosz, Przemysław Bąbelewski, Antoni Szumny, Zdenek Zadák, Anna Jankowska-Mąkosa, Damian Knecht

**Affiliations:** 1 Institute of Animal Breeding, Wroclaw University of Environmental and Life Sciences, 51-630 Wrocław, Poland; anna.jankowska-makosa@upwr.edu.pl (A.J.-M.); damian.knecht@upwr.edu.pl (D.K.); 2 Department of Pharmacognosy and Herbal Medicines, Wrocław Medical University, 50-556 Wrocław, Poland; piotr.okinczyc@umw.edu.pl; 3 Laboratory of Tissue Culture, Botanical Garden, Faculty of Biological Sciences, University of Wrocław, 50-234 Wrocław, Poland; jakub.szperlik@uwr.edu.pl; 4 Department of Food Chemistry and Biocatalysis, Wroclaw University of Environmental and Life Sciences, 50-375 Wrocław, Poland; bogdan.jarosz@upwr.edu.pl (B.J.); antoni.szumny@upwr.edu.pl (A.S.); 5 Department of Horticulture, Wroclaw University of Environmental and Life Sciences, 50-363 Wrocław, Poland; 6 Department of Clinical Biochemistry and Diagnostics, Charles University, Faculty of Medicine and University Hospital, 500 05 Hradec Králové, Czech Republic; zdenek.zadak@fnhk.cz

**Keywords:** *Ilex* sp., volatile compounds, PCA, correlation matrix, HD-SPME-GC/MS

## Abstract

Species of *Ilex* genus are particularly rich in bioactive constituents such as polyphenols, saponins, and alkaloids. In terms of phytochemical research, volatile compounds have not been as widely investigated as non-volatile ones. Therefore, in the present research we investigated the phytochemical profile of selected *Ilex* species by headspace-solid phase-microextraction coupled with gas chromatography-mass spectrometry (HD-SPME-GC/MS). Phytochemical profile between the same and different species was variable. For this reason, it was investigated by principal component analysis (PCA), matrix correlation (calculation of R^2^ and Pearson correlation parameter between samples) as well as hierarchical clustering (dendrogram). In our results, we exhibited that the amount of (*Z*)-2-hexenal and methyl salicylate was the most important factor for ascertaining similarity between samples. However, concentrations of some of these components were insufficient to describe all dependencies between *Ilex* specimens. Further analyses (PCA and dendrogram), exhibited that thymoquinone had secondary impact on phytochemical profiles similarity, as did *o*-methyl-anisole, (*E*)-2-decenal, salicylaldehyde and (*Z*)-3-hexen-1-ol. Differences of profile between samples may result from many factors such as local environmental conditions, creation of different chemotypes or even infestation by unknown pathogens or the presence of non-pathogenic microorganisms. Further research is required to investigate this phenomenon. Moreover, it was found that some of *Ilex* species may be potential sources of bioactive volatile components.

## 1. Introduction

Hollies are shrubs and trees of the monogeneric family *Aquifoliaceae*, in the genus *Ilex* L. specifically. With 669 *Ilex* species described in the literature, it is probably the second largest genus in the plant kingdom, right after *Diospyros* L. [1]. It is accepted in the literature that the genus most likely originated in Southeast Asia and later spread to other mesic habitats, achieving near cosmopolitan status [1]. Among the many uses of *Ilex* species, the widest known is its use in preparation of yerba mate, which is widely used in South American countries as a stimulant to reduce fatigue and as a tonic. However, various health benefits of mate have also been reported in the literature, especially its hepatoprotective, hypocholesterolemic, anti-inflammatory, antioxidant, as central nervous system stimulant, as well as antithrombotic and antirheumatic properties [2]. Such properties result from a variety of bioactive chemical compounds found in *Ilex paraguaiensis*, and extracted during the preparation of the beverage, namely polyphenols, among them chiefly various chlorogenic acids, xanthines like caffeine and theobromine, flavonoids, amino acids, vitamins and minerals. There is, however, a health risk concerning mate consumption, as it may contain polycyclic aromatic hydrocarbons [3]. Other *Ilex* species used to make teas are found in China, such as *I. latifolia* and *I. kaushe*. Those, however, do not contain caffeine, which may limit their marker appeal. There is a tradition of using *Ilex* species in medicine, especially in China and South America. In China *I. chinensis* and *I. cornuta* were widely used since at least the 16th century; however, there is very little data on their actual pharmacological effectiveness. There is some literature on *I. brevicuspis* and *I. guayusa*, but the lion’s share of articles concentrates on the pharmacological properties of *I. paraguaiensis* [1]. Other species, such as *I. aquifolium* in Europe, have been used as ornamental plants and a source of timber [1]. Another species used as an ornamental plant, but also as a food source and medicine, is *I. perneyi*, also found in China [4]. Other hybrid *Ilex* species used as an ornamental plant and recently in food processing is *I. meserveae* [5].

Considering the great diversity of *Ilex* species and their diverse uses, only a few of them have been mentioned in this paper together with their known bioactive non-volatile compounds. It is surprising how few inquiries into volatile fractions found in *Ilex* species have been published. Of those, nearly all concentrate on *l. paraguaiensis*, while other species remain virtually terra incognita. This is quite surprising, as many of those species have pharmacological or at least folk medicine uses [1]. Among volatile compounds identified in *I. paraguaiensis* were hydrocarbons like dodecane, tridecane, tetradecane, pentadecane, monoterpens: myrcene, linalool, β-cyclocitral, geranial, nerol, geraniol and geranyl acetate, norisprenoids: β-damascenone, α-ionone, β-ionone, as well as carbonyl compounds: 6-methyl-5-hepten-2-one, (*E*,*Z*)-3,5-octadien-2-one, aldehydes: nonanal, (*E*,*Z*)-2,4-heptadienal, (*E*,*E*)-2,4-heptadienal, (*Z*)-2-decenal, (*E*)-2-decenal, (*E*,*E*)-2,4-decadienal and alcohol: 1-octanol, to mention the more abundant ones [6]. There are two main methods used to analyse volatile compounds: Likens–Nickerson and headspace-solid phase micro-extraction (SPME), which are usually coupled with gas chromatography (GC) and mass spectrometry (MS) [7,8]. Likens–Nickerson is the older one, and in brief it allows for extraction of aromas through placing the sample in Likens–Nickerson apparatus, extracting it with solvents and the subsequent removal of solvents, which leaves pure volatile compounds [6]. SPME achieves similar effects in a different way, by heating and agitating the sample in the presence of previously desorbed SPME fibre, which absorbs aromas released from the sample. Those can be released from the fibre by heating. Usually, volatile compounds sourced thusly are later analysed by GC-MS, which allows for their precise identification and quantification [3,9].

We aim to analyse rarely researched species of the *Ilex* genus which are available to us and establish their volatile compound profiles and potential use. Among those we selected varieties of *Ilex meserveae*, *Ilex aquifolium*, *Ilex altaclariensis* and *Ilex perneyi*. All of those varieties, as well as their parent species, all are possessed of evergreen leaves, albeit they vary greatly in growth strength, shape and colour of leaves, which are often notched and spiky, with wavy edges. *Ilex aquifolium* is the most common choice for ornamental plants, due to its dark green, leathery, shiny leaves. We analysed three varieties: *Ilex aquifolium* ‘Alaska’ female variety, very valuable due to the glossy leaves, slow growth and high resistance to low temperatures up to −29 degrees Celsius; *Ilex aquifolium* ‘Pyramidalis Aurea Marginata’ variety with yellow-colored leaves margins with dense, pyramidal growth; and *Ilex aquifolium* ‘Argentea Marginata’ shrubs with yellow-coloured leaves margins and red fruits. The latter is also known as ‘Argenteamarginata’. The second most common in cultivation is *Ilex meserveae*, a hybrid of *Ilex aquifolium* and Japanese *Ilex rugosa*. We analysed *Ilex × meserveae* ‘Blue Girl’ female variety characterized by a dense growth and red fruit; *Ilex × meserveae* ‘Blue Angel’ female slowly growing variety with red fruits; and *Ilex × meserveae* ‘Mesgolg’ characterized by hard, broadly elliptical nearly flat leaves, of sharp top and cuneatic base, with serrated margins. It has a dark green top of the leaf blade, and a light green bottom with a blue tone. A variety form lemon-yellow fruits. The last among those was *Ilex × meserveae* ‘Mesan’ male variety with hard, oval or broadly elliptical evergreen leaves, with serrated margins, a glossy top and light green bottom of the leaf blade. We have also included *Ilex × altaclarensis* ‘Lawsoniana’, a hybrid between *Ilex aquifolium* and *Ilex perado*, occurring in Madeira, Canary Island and Azores; and *Ilex perneyi* coming from Mongolia and North-East China, where it forms high shrubs or low trees up to 7 m [10,11].

Volatile compounds play a significant role in plant defense mechanisms, food preservation, and the fragrance industry. They are essential for plant communication and interaction with the environment, contribute to the aroma and scent properties of food, and have both positive and negative environmental impacts [12]. So far, our research has focused on polyphenolic compounds, saponins, and fatty acids contained in *Ilex* sp. [11,13,14,15,16]. In order to better understand and learn more about the *Ilex* species, the aim of our research was analysis of *Ilex* volatile components. We hope it will allow us to establish how similar or dissimilar their volatile fractions are, which in turn could inform future research regarding their potential applications, as well as shed some light on their metabolism.

## 2. Results

### 2.1. HD-SPME-GC/MS Profiles of Ilex spp. Volatile Components

Results of headspace-solid phase microextraction coupled with gas chromatography and mass spectrometry (HD-SPME-GC/MS) analyses of *Ilex* spp. volatile component profiles were presented in Table 1 (HD-SPME-GC/M profile of *Ilex* spp. volatile compounds). Components were identified by a comparison of their GC-MS spectra and retention indexes to those of the literature. A total of 39 components were identified. Among them, only two components had unclear identification.

Generally, in all samples three main groups of components were found—non-terpenes aliphatic components, non-terpenes aromatic derivates and terpenes and terpenoids. No singular component dominated HD-SPME-GC/MS profiles of volatile components of investigated cultivars (concentration > 50%). Most of them exhibited rather mixed composition of different numbers of components. In most cases, their concentration usually did not exceed 30%.

Non-terpenes aliphatic components took from 9.43% (I.pe.M) to 83.96% (I.aq.F6) of all peaks area. Most samples had around 40% of those compounds. This group was mainly composed of alcohols (from 2.01% to 36.57%) and ketones aldehydes with ketones (from 7.42% to 76.39%). Among all non-terpenes aliphatic components, the most prevalent derivative was (*Z*)-2-hexenal. It was observed in every cultivar, with concentrations ranging from 0.28% to (I.pe.M) to 73.8% (I.aq.F6). The next most concentrated non-terpene aliphatic compounds were (*E*)-2-decenal (the highest concentration in I × alt.F) and 3-hexen-1-ol (the most abundant in I × m.F3).

The second considerable group of components was non-terpenes aromatic derivates. It was composed of mainly esters of benzoic acid (ex. methyl salicylate) and its derivatives (ex. salicylaldehyde), as well as other types of substances (ex. p-methyl anisole). It was the dominant group of components in four samples (I.aq.F2, I.aq.F5, I × m.F3 and I.pe.M). The most important component of this group was methyl salicylate, which was observed in almost all samples except I.aq.F6. The highest concentration of methyl salicylate was observed for I.pe.M (72.83%). It was also a major component of I.aq.F2 and I.aq.F5. Apart from methyl salicylate the remainder of non-terpenes aromatic derivates did not show considerable presence and were rather minor components (concentration < 10%).

The last group of components were terpenes and terpenoids. In comparison to previous groups of components, terpenes and terpenoids had limited presence in HD-SPME-GC/MS profile of most cultivars. It was the main group of components for only two samples—I.aq.F4 (13.74%) and I × m.F1 (25.64%). Both cultivars had thymoquinone as the main component. Most terpenes and terpenoids had a concentration under 5% among all samples. Apart from thymoquinone only *p*-cymene, limonene and isolongifolene had concentrations above 5% in some cultivars.

### 2.2. Statistical Analysis

#### Principal Component Analysis (PCA)

**Figure 1:**
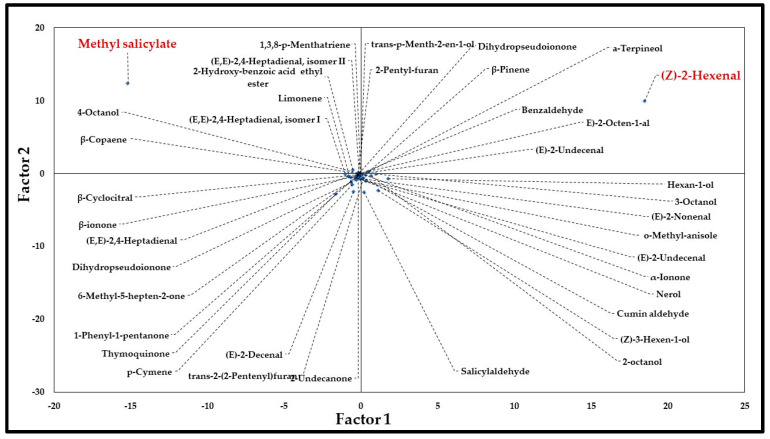
Projection of the variable on the factor plane (two-factors model).

**Figure 2:**
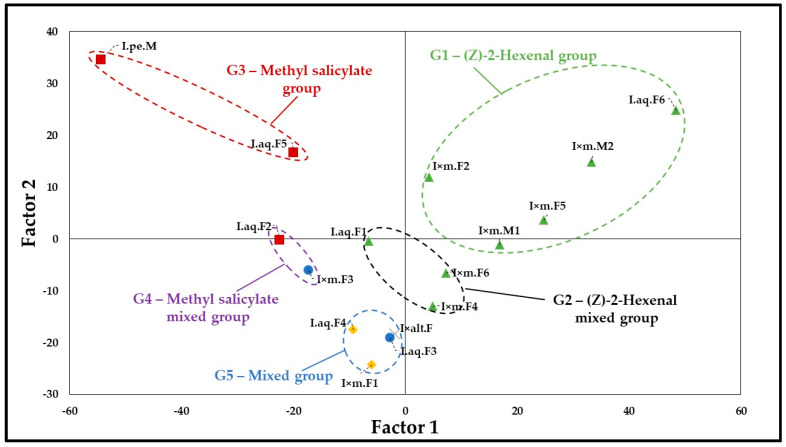
Projection of the cases on the factor plane (two-factors model). Legend: Main component of samples as shown: Green triangle—(*Z*)-2-Hexenal; Red square—Methyl salicylate; Blue point—(*Z*)-3-Hexen-1-ol; Yellow diamante—thymoquinone; Black cross—2-Decenal.

Results of PCA are shown in Figure 1 (Projection of the variable on the factor plane) and Figure 2 (Projection of the cases on the factor plane). PCA exhibited that a two-factor (two principal components) model exhibited 77.4% of quality representation. The three-factor model had 83.8% and four-factor 89.7% of quality representation. Therefore, quality of representation in two-factor model was over 75% and the next models changed this value for only around 5–6%; two-factor model was found as sufficient to present a graphical representation of samples distribution. Presentation of three-factors model (3D graph) may lead to misinterpretation, as factor 3 exhibited considerably lower impact on quality of representation than factor 1 and factor 2. However, due to the complex composition of some samples, reliable analysis required inclusion of a three-factor model (see scree plot in Supplementary Data—PCA-3 factor model). Scree plot showed that the three-factors model had the sharpest line change.

In three-factors model, factor 1 included 51.94% equivalents of covariance matrix, factor 2 had 25.48% and factor 3 only 6.41%. The highest impact on factor 1 exhibited two components: (*Z*)-2-hexenal (58.3% of variable contribution) of and methyl salicylate (39.5% of variable contribution), respectively. A similar situation was observed for factor 2; however, methyl salicylate had a stronger impact (52.9% of variable contribution) than (*Z*)-2-hexenal (34.4% of variable contribution). Factor 3 proved more complex than factor 1 and factor 2. The highest impact on its variable contributions was shown by thymoquinone (28.3%), p-methyl anisole (23.4%), (*E*)-2-decenal (19.4%) and salicylaldehyde (12.8%). Contributions of remaining components were under 5%.

Generally, samples may be divided into five groups (clusters) in Figure 2. Group 1 was composed of I.aq.F6, I × m.M2, I × m.F5, I × m.M1 and I × m.F2. Concentration of (*Z*)-2-hexenal above 30% was the main criterion of samples similarity. For this reason, Group 1 was named “(*Z*)-2-Hexenal group”.

Group 2 contained I × m.F6, I × m.F4, and I.aq.F1. The main component of this group was also (*Z*)-2-hexenal, however, it varied from 20.86% to 17.11%. Moreover, inside group 2 was observed also the considerable presence of other components (from 9.25% to 15.88%). (*Z*)-2-Hexenal was the major component of Group 2, but not so dominant such as in Group 1 and therefore the second cluster was described as “(*Z*)-2-Hexenal mixed Group”.

Group 3 was composed of I.aq.F5 and I.pe.M and it contained methyl salicylate as its major component (concentration > 30%). Due to the dominance of methyl salicylate inside third cluster it was named “Methyl salicylate group”.

Group 4, similar to the last group, also contained significant presence of methyl salicylate, but not so strong (>20% and >25%) as cluster 3. This group included I × m.F3 and I.aq.F2. Apart from methyl salicylate, it was also rich in others components and methyl salicylate was not always the dominant component. For this reason, the fourth cluster was named “Methyl salicylate mixed group”.

The last cluster (Group 5) was composed of four samples (I × alt.F, I × m.F1, I.aq.F4 and I.aq.F3). Characteristics for this group included the lack of a significant amount of (*Z*)-2-hexenal nor methyl salicylate (anyone < 10%). Their samples contained 2-decenal, thymoquinone and 3-hexen-1-ol as dominant substances. For this reason, the fifth cluster was named “mixed group”.

In summary, factor 1 and factor 2 had shown the most significant impact on graphical presence groups 1, 2, 3 and 4. (*Z*)-2-hexenal and methyl salicylate in most samples were found to exercise a crucial impact on these two factors. Factor 3 was important to distinguish group 5 from the other groups, caused by the significant presence of thymoquinone and 2-decenal. Factor 3 also served to delineate groups 2 and 4 from main groups 1 and 2.

### 2.3. Correlation Matrix Heat Map and Hierarchical Clustering Analysis (Dendrogram)

**Figure 3:**
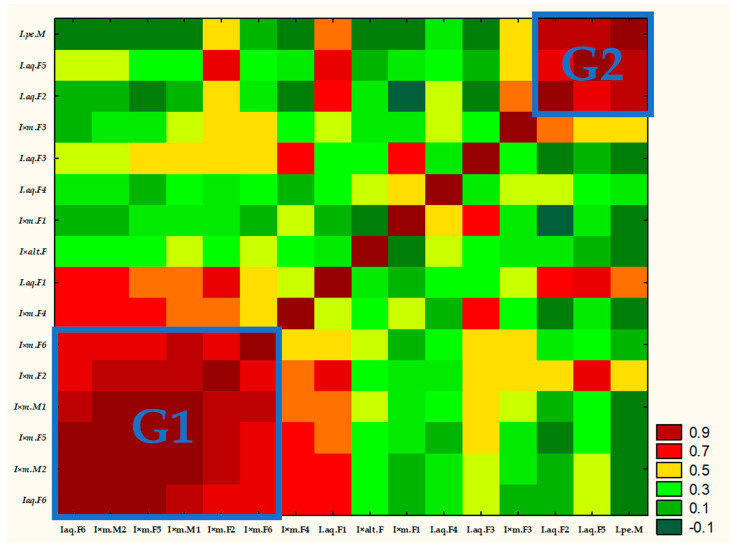
Heat map for R2 parameter of correlation matrix.

Results of correlation matrix between *Ilex* spp. are presented in Figure 3 as a heat map. It was built of R2 parameters and arranged in terms of correlation. Full results of correlation matrix analyses are attached as Supplementary Data. Analysis has shown that most of the samples correlated with at least three samples (*p* < 0.05). In most cases the R2 parameter was also positive, which showed positive correlation.

According to the heat map for R2 parameters it was possible to obtain two main groups. The R2 parameter for these groups was above 0.7 or higher. The first and bigger group contained six samples: Iaq.F6, I × m.M2, I × m.F5, I × m.M1, I × m.F2 and I × m.F6. The concentration of 2-Hexanal was above 20% for all samples in this group. The second group consisted of I.aq.F2, I.aq.F5 and I.pe.M, with a concentration of methyl salicylate above 24%. However, samples inside G1 (I × m.F2) and G2 (I.aq.F5) also correlated with the R2 parameter 0.709 due to significant concentration of methyl salicylate (>19%). Moreover, samples inside the G1 and G2 clusters were also correlated with samples outside both clusters.

**Figure 4:**
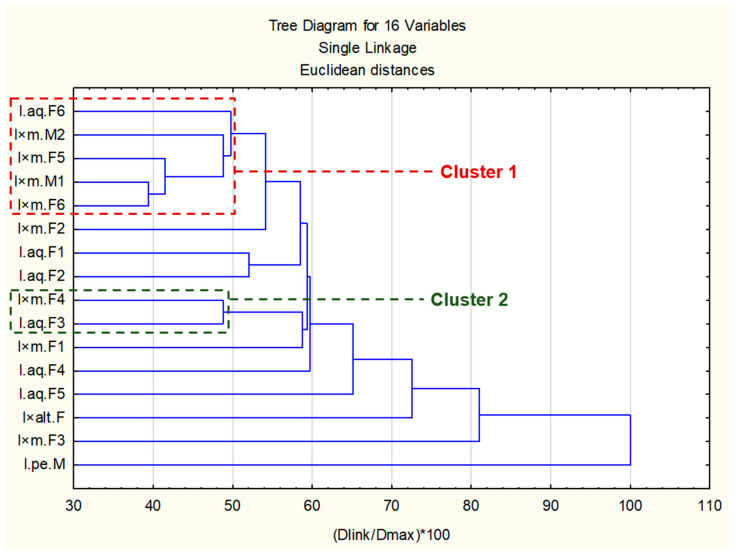
Dendrogram of *Ilex* sp.

As we found it impossible to establish a clear dividing line in the heat map (based on (*Z*)-2-hexenal and methyl salicylate concentration), hierarchical cluster analysis was used to better track relationships between samples (Figure 4).

As shown in the dendrogram, most samples did not create simple cluster groups. Only two simple clusters were possible to create among all samples. Single linkage under 50% of Euclidean distance was created between them.

Cluster 1 was composed of five specimens (I.aq.F6, I × m.F5, I × m.M1 and I × m.F6). It was characterized by presence of components rich in 2-Hexanal (above 30%) so it partially corresponded to PCA G1 and PCA G2 as well as heat map G1 groups.

Cluster 2 contained I.aq.F1 and I × m.F4. Specimens which grouped in this cluster had similar concentrations of (*Z*)-3-Hexen-1-ol (around 12–13%), salicylaldehyde (around 10–15%) and some minor components (>5%).

Other clusters were created above 60% of Euclidean distance and they contained cluster 1 or 2 or both of them. Moreover, the composition of specimens’ volatile components in these potential clusters was not as similar as in clusters 1 and 2. It was also not possible to create any cluster of samples with a strong presence of methyl salicylate, in contrast to PCA. These results showed that the presence of methyl salicylate was not so common as 2-Hexanal and may be connected to specific metabolic pathways. However, it is necessary to perform follow-up investigations to prove this hypothesis.

## 3. Discussion

Plant volatile compounds (VOCs) are a diverse class of components that play equally diverse roles. In effect, they encompass volatile compounds produced by plants that affect growth and metabolism of plants, bacteria, fungi and animals, but also bacteria and fungi produced compounds that affect the growth and development of plants through both allelopathy and interaction with hormone regulation. It is worth noting that numerous VOCs take part in the stress response of plants [12]. Most of the VOCs are in essence various terpenoids produced both in leaves and in the roots of plants, although the latter are less well understood. There are over 30,000 known terpenoids, divided into hemiterpenes (C5), monoterpenes (C10), and sesquiterpenes (C15), which makes them the most numerous, as well as the most diverse group of plant compounds. Many of these number are, in fact, volatile compounds [12].

To group samples in clearly distinguishable groups of plants related by chemical profiles, principal component analysis (PCA) was performed. PCA has shown that two-factor (two principal components) model covered 77.4% of quality representation, which was deemed sufficient. Two components were found to have a dominant impact on factor 1: (*Z*)-2-hexenal and methyl salicylate, with the remaining components showing much less significant impact. A similar situation was observed for factor 2, however, methyl salicylate had a stronger impact than (*Z*)-2-hexenal. Secondary impact on classification in the 3-factor model was exhibited also by thymoquinone, *o*-methyl anisole, (*E*)-2-decenal and salicylaldehyde. This allowed for the division of all samples into five clusters in PCA analysis, according to the criteria described in the Methods section. Moreover, hierarchical clustering analysis (dendrogram) established that (*Z*)-3-hexen-1-ol the shows the second most important impact on *Ilex* spp. phytochemical classification of volatile components. Therefore, we will try to illuminate the potential biological significance of those divisions.

(*Z*)-2-Hexenal was found in significant quantities in almost all analyzed specimens, ranging from 7.7% in L.aq.F3 to 73.8% in L.ag.F6. Samples in which concentrations of any compound did not reach 5% were classified as not containing notable amounts of it. Only in I.aq.F2, I.aq.F4, I × m.F3, I × m.M1 and I.pe.M was the concentration of (*Z*)-2-Hexenal lower than that, which makes it the most ubiquitous compound of all of the ones we analyzed. This is not surprising, considering its diverse functions in plants. Those could be broadly divided into plant defense, communication, and regulatory roles. (*Z*)-2-Hexenal is involved in hydrogen peroxide burst in plants at the plasma membrane and directed flow of calcium ions, which are part of early signaling events in plants. Their activation contributes to plants’ resistance to insect attacks, as demonstrated in *Arabidopsis thaliana* when challenged with the diamondback moth [17]. (*Z*)-2-Hexenal was also reported to disrupt cell membrane composition of microorganisms and in doing so prevent pathogenic infection of plants, as well as food spoilage [18]. (*Z*)-2-hexenal was applied in different fields as well due to its antimicrobial properties and its role in flavor and fragrance enhancement. Its antibacterial properties are used in fruit processing, effectively inhibiting growth of bacteria and improving shelf-life [19]. Other important roles lie in plant communication and stress response, as (*Z*)-2-Hexenal was reported to play a role in plant-to-plant and plant-to-insect communication, signaling distress and triggering defense responses in neighboring plants. Part of its role is as one of the Green Leaf Volatiles (GLVs) which are emitted by plants in response to wounding or stress [20]. Additionally, it activates chloroplast degradation in tomatoes, which results in color change during ripening. This is achieved by activation of lipoxygenase (LOX) pathway, which decomposes free fatty acids [21]. All of the above points to *Ilex* species having the potential for being used as a source of antimicrobial or preservative formulations. Particularly suited to this role would be the species from (*Z*)-2-Hexenal cluster from our PCA analysis, so I.aq.F6, I × m.M2, I × m.F5, I × m.M1 and I × m.F2, as all of those species contained more than 30% of (*Z*)-2-Hexenal in their volatile fraction. Plants from the *Ilex* genus are known for their antimicrobial properties which confirms our previous study by Paluch, Okińczyc et al. (2021) [14]. Extracts significantly reduced the biofilm viability of *S. aureus*, suggesting potential use in antiseptics and diuretics. Based on research we can assume that VOCs of *Ilex* sp. may also play an important role in antimicrobial activity of the whole extract and be used in the pharmaceutical or cosmetic industry.

Methyl salicylate was the second most important of the compounds we found in analysed species, albeit it exceeded 5% of content in only five species, from 14.49% in I.aq.F1 to 73.83% in I.pe.M. This is significant, because methyl salicylate is involved in many important processes concerning plant defense, stress resistance and signal transduction in plants. Perhaps its most important role lies in activation of systemic acquired resistance, as was demonstrated in *Arabidopsis*, and as a signal molecule transported through phloem it could induce de novo synthesis of salycylic acid in distant tissues [22,23]. This signal can be also transported as a volatile emission through the air, inducing systemic acquired resistance in distant plants [24]. Additionally, methyl plays an important role in reactions to both abiotic and biotic stresses. Chiefly, it can increase plant heat tolerance, albeit accumulation in leaves can also decrease it by altering antioxidant defenses [25]. Methyl salicylate has also a noteworthy role in the defense against both herbivores and microbial pests. In this it acts more as a deterrent than a toxin, eliciting changes in plant volatile compound profiles that can both deter herbivores like spider mites and attract beneficial predatory mites [26]. This points to the plants forming a methyl salicylate group from our PCA analysis as a potential source of both plant defense stimulators and herbivore repellents, as both I.aq.F5 and I.pe.M contained more than 30% of methyl salicylate in their volatile fraction. Such an application finds precedent in studies conducted by Gao et al. (2022, 2025) [27,28] on the effect against *Haemaphysalis longicornis* using *I. purpurea* and *I. chinensis* which showed significant acaricidal activity against unfed larvae, nymphs and adults. The main active substance identified in his research was methyl salicylate (*I. purpurea* 77.90% and *I. chinensis* 85.65%). As for our results, I.pe.M had the highest methyl salicylate content—72.83%, which may indicate that this *Ilex* sp. may also have anti-tick properties.

(*Z*)-3-Hexen-1-ol, commonly referred to as leaf alcohol, is a significant green leaf volatile in many plants. It was present in significant amounts in a volatile fraction of I × m.F3 (25.95%). It is responsible for both direct and indirect defense mechanisms in plants, as it can induce the expression of defense-related genes responsible for synthesis of jasmonic acid (JA) and ethylene (ET), which are crucial for activating defense responses against herbivores [29,30]. It can also be converted into its glycosides, which are involved in enhancing insect resistance [31,32]. Acting indirectly, it can induce emission of blends of volatile compounds by plants, which in turn attract natural enemies of herbivores [33], as well as act as a signaling compound inducing systemic acquired defense in nearby plants [34]. Additionally, it has been reported to enhance drought tolerance in tea plants subjected to cold stress through mediating reactive oxygen species and stomatal conductance, which is achieved through modulation of absicinic acid homeostasis [33].

O-methyl anisole, also known as 1,2-dimethoxybenzene or veratrole is an important volatile compound regarding floral scent and pollinator attraction. It was found chiefly in volatile fractions of I × m.F3 (21.7%) and I × m.F6 (14.54%). O-methyl anisole is an important component of the floral scent in many plant species, including roses and *Silene* *latifolia* [35,36]. As such, it plays a key role in attracting pollinators, for example the moth *Hadena bicruris* for *Silene latifolia* [36]. Among analyzed species, I × m.F3 showed a significant amount of o-methyl anisole (21.7%) in its volatile fraction. It is worth noting that the presence of (*Z*)-3-Hexen-1-ol, O-methyl anisole and methyl salicilate in roughly similar amounts (c. 20%) designates I × m.F3 as a possible source of useful metabolites of antimicrobial, anti-herbivore and also pollinator attractive properties, which combines significant usefulness in a singular plant, as well as puts it as the basis of Methyl salicylate mixed group as described in our PCA analysis. For I.aq.F2 situation is far less clear, as while it contains close to 25% of methyl salicylate, the remainder of its chemical composition does not form any clear pattern of useful metabolites.

Hexane-1-ol plays an important role in plant defense mechanisms, plant-to-plant signaling and in regulation of the synthesis of plant volatile compounds. It has been found primarily in I × m.F6 among the species we analyzed (15.88%). It is one of the green leaf volatiles (GLVs) that are emitted when plants are damaged by herbivores [37]. One such example is its interaction with odorant-binding proteins (OBPs) in insects, which are crucial for host plant location. For example, in the alfalfa plant bug, hexane-1-ol binds to OBPs and acts as a repellent, influencing the insect’s behavior and potentially reducing herbivory [38]. Together with (*Z*)-2-Hexenal and o-Methyl anisole, hexane-1-ol forms the triad of dominant components among VOCs of I × m.F6; however, only the latter exceeds 20%. It would suggest that plants from this group may combine antimicrobial, anti-herbivore and flavouring properties in a potentially useful way, albeit it is less obvious for other members of (*Z*)-2-Hexenal mixed Group, as I × m.F4 lacks any clear dominant compound except the group’s namesake, while I.aq.F1 contains a significant amount of methyl salicylate, which could change its properties.

Thymoquinone is a bioactive compound primarily found in the seeds of the plant *Nigella sativa*, commonly known as black cumin. It was also found in I × m.M1 (25.61%). It is highly interesting, as it is a compound possessing known therapeutic and bioactive properties, chiefly antioxidant and anti-inflammatory, which is useful in some liver disorders as well as arthritis and asthma [39,40,41], mostly through activation of antioxidative enzymes such as superoxide dismutase (SOD), catalase (CAT), and glutathione peroxidase (GPX4), which help in reducing reactive oxygen species (ROS) and protecting cells from oxidative damage [42,43]. On top of that, it is also reported to possess anticancer properties through inhibition of cell proliferation, inducing apoptosis, preventing angiogenesis and a metastasis in various cancer types, including breast, lung, prostate, and pancreatic cancers [41,44,45,46,47,48,49,50,51]. It has also been shown to inhibit the growth of Aspergillus flavus and reduce its toxin production when combined with infrared radiation [40]. It suggests that I × m.M1 may be useful in previously unexplored applications in natural therapies, in medicine and veterinary medicine.

(*E*)-2-Decenal is a volatile organic compound that plays a significant role in plant defense mechanisms, particularly against fungal pathogens. It has been found in I × alt.F in a significant amount (27.95%). It has been shown to inhibit the growth of the plant pathogen *Alternaria alternata*. This was achieved through multiple mechanisms, including disruption of fungal cell membrane integrity, induction of reactive oxygen species (ROS) accumulation, and resulting oxidative stress. Those caused hyphal abnormalities, reduced spore production, and suppressed spore germination. Disruption of cell membranes increased leakage of DNA and soluble proteins, which further contributed to its antifungal efficacy [52]. I × alt.F was assigned to the Mixed Group of PCA analysis, as neither (*Z*)-2-Hexenal nor methyl salicilate were dominant in it. A similar situation occurred for I × m.F1, I.aq.F4 and I.aq.F3. All those species lacked a clearly dominant compound in their profile, so it is difficult to formulate an opinion as to their potential uses, other than the promising antifungal activity of I × alt.F.

All of the properties of the compounds described above are highly interesting, as they open the way to the application of biostimulants, antimicrobiological agents and biopesticides. Another interesting avenue is the application of VOCs as stress alleviation agents, which could be very useful in unstable climatic conditions and resulting multi-stress (drought, high temperature, pathogens) in agricultural plants. Plant VOCs play a role in plant–animal interactions as well, as they both attract pollinators and repel herbivores. Pollinator attractants are mainly found among VOCs of small molecular weight and high vapor pressure. Both of those traits are essential for effective diffusion in the environment and thus fulfil the function of attractant. This is usually accompanied in plants by the production of sweet nectar, which serves as a reward for pollinators [53].

Additionally, volatile compounds play an essential role in food processing and influence the taste, aroma, and overall quality of food products. These compounds are indicators of food quality, shelf life and authenticity, and their analysis is essential to optimize production and ensure consumer satisfaction [54]. The scientific literature confirms that Ilex species offer several potential benefits for skincare and cosmetics formulations, including antioxidants, anti-inflammatory and collagen-promoting properties, as well as a pleasant aroma. Considering the number of patents related to plants of the Ilex species, we can distinguish applications in pharmaceutical, nutraceutical, food processing, and supplements, followed by the cosmetics industry [55]. In the light of the compounds found in our samples, it may even possibly add anticancer applications to this significant list in the form of thymoquinone found in I × m.M1.

## 4. Materials and Methods

### 4.1. Plant Material

Trees and shrubs were sourced from a collection located in Psary near Wrocław, from the Research and Training Station of Vegetables and Ornamental Plants–Psary of the Faculty of Life Sciences and Technology of the Wrocław University of Environmental and Life Sciences. There are around 1000 trees, shrubs and perennials in the collection, which are used mainly as teaching aids for students. Among them there are shrubs of the genus *Ilex* sp., planted on a clay-sandy soil. Before planting plants, the soil was enriched with acidic peat at a dose of 20 L per 1 m^2^ to improve the physical and chemical properties of the soil, i.e., porosity for water and air and lowering pH to pH 5.5 from native pH of 6.7. The soil in the arboretum belongs to class III clay soils included in the so-called degraded chernozems. The soil was mulched with shredded pine tree bark c. 5 cm deep to provide constant soil humidity that is beneficial for *Ilex* sp., as well as to inhibit weed growth. Samples were sourced from c. 10-year-old bushes grown under a continuous high canopy of old trees. Besides teaching purposes, the collection is also utilized as a place for scientific research and acclimatization of selected tree taxa. Plant material used in the study is presented in Table 2.

Plant material in the form of shoots with leaves, was sourced from annual growths collected from the bushes in a way that the cuttings were distributed evenly on the range of the entire crown both from the northern, southwestern and eastern sides evenly. 10–20 cm long branches were cut from annual increases. Then the leaves were separated from the stems and air dried. The cuttings were sourced on 4 May 2017.

### 4.2. Chemical Composition of Samples

The determination of volatiles present in leaves of *Ilex* species was conducted by solid-phase microextraction (SPME) using a 50/30 μm divinylbenzene/carboxen/polydimethylsiloxane fiber (Supelco, Bellefonte, PA, USA) with a length of 1 cm. Five grams of plant material were ground in a mortar after the addition of 2-undecanone as an internal standard (5 μg suspended in 100 μL of deionized water) and placed in 5-mL glass vials sealed with plastic screw caps and Teflon-coated septa. After conditioning the SPME fiber at 270 °C, it was exposed to the headspace volatiles at 60 °C for 30 min. Subsequently, the coated fiber was removed from the headspace and injected at 230 °C into a Varian Chrompack CP-3800 system equipped with a Saturn 2000 GC/MS/MS mass analyzer and a Zebron DB-1 standard non-polar column (30 m length, 0.25 mm diameter, 1 μm film thickness, Varian, Walnut Creek, CA, USA). Helium was used as the carrier gas at a flow rate of 1 mL/min. The initial column temperature of 40 °C was held for 1 min, followed by a temperature gradient of 2 °C/min until reaching 60 °C, after which the gradient increased by 5 °C/min until completion of the analysis at 250 °C. The identification of individual components was performed using the retention index system (RI) according to NIST23, employing chromatographic runs of a homologous series of n-alkanes for calibration.

### 4.3. Statistical Analysis

Statistical analysis was performed by Statistica 14.0.0.15 software (Tibco Sofware Inc., Palo Alto, CA, USA). Tests included principal components analysis (PCA), correlation matrix analysis (presented as heat map) and hierarchical cluster analysis (as dendrogram).

Data input for all tests was a percentage value of total peaks area of SPME chromatogram. Substances of at least 1% of the relative area (in any sample) were used to construct the matrix. In PCA analysis, components were used as variables, while *Ilex* samples were cases. Analysis based on covariances (as SS/(N − 1)) and components were used as variables.

Correlation matrix analyses included calculation of R^2^ as well as Pearson correlation parameter between all samples. *Ilex* spp. were used as variables. Heat map for correlation matrix was prepared from calculated R^2^ parameter.

## 5. Conclusions

This work has provided, for the first time, important data concerning the differences in the volatile composition of the *Ilex* species. Using HS-SPME-GC/MS analysis, significant variability was observed both within and between *Ilex* species. Statistical evaluations, including PCA, matrix correlation, and hierarchical clustering, revealed that (*Z*)-2-hexenal and methyl salicylate were the primary compounds influencing sample similarity, while thymoquinone, o-methyl-anisole, (*E*)-2-decenal, salicylaldehyde, and (*Z*)-3-hexen-1-ol contributed secondarily to the observed variation. These compositional differences likely reflect the influence of environmental factors, the existence of distinct chemotypes, or interactions with pathogenic and non-pathogenic microorganisms. Overall, the findings highlight the potential of certain Ilex species as valuable sources of bioactive volatile constituents and emphasize the need for further research to better understand the ecological and biochemical factors shaping their volatile profiles. As we learn more about the composition and properties of these plants, new possibilities for their potential use are emerging. Further scientific research is needed to better understand the mechanisms of action of *Ilex* sp. and confirm their positive health effects and potential application.

## Figures and Tables

**Table 1:** HD-SPME-GC/M profile of *Ilex* spp. volatile compounds.

Compound	ERI	LRI	I.aq.F1	I.aq.F2	I.aq.F3	I.aq.F4	I.aq.F5	I.aq.F6	I × m.F1	I × m.F2	I × m.F3	I × m.F4	I × m.F5	I × m.F6	I × m.M1	I × m.M2	I × alt.F	I.pe.M
(*Z*)-2-Hexenal	830	827	15.32	2.95	7.70	4.16	16.30	73.80	32.68	30.96	3.56	17.11	41.40	20.86	4.70	55.24	9.27	0.28
(*Z*)-3-Hexen-1-ol	845	839	1.31	0.05	11.03	0.31	0.67	4.15	8.70	9.66	25.95	12.68	11.74	5.74	15.03	6.91	1.97	0.33
Hexan-1-ol	860	854	2.75	1.76	10.03	0.76	0.61	2.63	9.12	10.85	1.63	1.85	10.43	15.88	3.89	3.07	2.01	0.41
* 2,4-Hexadienal isomer I	872	n.d.	2.38	1.40	0.02	0.20	0.01	0.00	0.34	0.24	0.65	3.41	0.17	0.24	4.04	0.70	0.19	0.04
2,4-Hexadienal isomer II	882	877	0.67	0.06	0.54	0.03	0.04	0.88	0.69	0.15	0.14	0.58	0.84	0.31	0	0.19	0.50	0.02
2-Methyl-1-hexanol	915	917	0.04	0.04	0.04	0.20	0.04	0.02	0.00	0.00	0.07	0.06	0.01	0.07	0.02	0.00	0.03	0.01
Benzaldehyde	938	942	3.37	7.99	4.25	5.35	6.20	2.70	0.22	7.36	7.79	8.35	0.14	3.79	1.98	5.85	9.23	2.29
4-Octanol	968	972	0.00	0.09	0.55	0.35	0.60	0.11	0.13	0.09	0.53	0.85	2.42	1.81	0.60	1.85	2.50	0.01
*β*-Pinene	977	973	0.16	0.05	0.06	0.07	0.01	1.79	0.04	0.06	0.13	0.17	3.24	1.11	0.07	0.55	0.02	0.17
6-Methyl-5-hepten-2-one	972	975	2.46	1.32	4.40	0.85	0.13	0.39	0.03	0.07	0.18	0.16	0.04	0.23	0.14	0.07	0.43	0.29
2,4-Heptadienal, isomer I	974	977	4.56	8.47	0.05	5.57	1.14	1.12	0.72	0.40	1.13	3.07	0.06	0.22	0.57	0.18	0.09	2.67
2-Pentyl-Furan	976	979	2.90	0	0.44	0.56	0.19	0.81	2.84	0.23	0.20	2.72	0	0.35	0.33	0.11	1.05	2.56
2,4-Heptadienal, isomer II	979	980	3.28	10.86	0.83	2.52	0.75	0	0	0.56	0.73	0.70	0.12	0.63	0.37	0.20	1.20	0
3-Octanol	978	982	1.04	0.32	1.12	0.62	0.91	0.55	4.08	0.28	1.00	1.16	5.09	2.33	1.00	5.50	2.84	0.19
*trans*-2-(2-Pentenyl)furan	983	985	0.59	0.73	0.02	3.16	0.14	1.03	1.26	0.03	0.57	8.45	0	0.65	0.31	0.03	0	0.07
2-Octanol	988	988	0.52	0.07	0.81	0.19	0.93	0.01	2.44	0.19	0.89	0.92	5.85	1.32	1.33	3.24	5.15	0.09
1-octanal	998	998	1.80	0.40	10.99	1.30	0	1.36	0	0.28	0.53	15.37	2.28	0	11.03	0.21	0	0.07
*o*-Methyl-anisole,	1004	1006	0.61	6.37	0	12.41	0.23	4.63	12.43	4.01	21.70	0	0	14.54	0	7.97	7.14	1.18
*p*-Cymene	1016	1015	5.88	1.36	5.27	2.75	1.36	0.02	1.17	0.48	1.09	1.00	1.30	0.22	10.82	0.29	1.06	0.95
Limonene	1022	1025	9.25	3.95	6.92	7.32	3.33	0.36	4.77	1.98	4.46	5.34	3.27	3.03	1.59	1.28	4.27	2.13
(*E*)-2-Octen-1-al	1032	1035	3.29	2.80	0.04	0.38	0.19	0.05	0.45	0.04	0.19	0.50	0.07	0.29	0.40	0.14	0.52	0.43
*trans*-Rose oxide	1112	1115	0.49	0.26	0.08	0.06	0.06	0.04	0.17	0.03	0.05	0.12	0.03	0.06	0.03	0.02	0.07	0.01
*trans*-*p*-Menth-2-en-1-ol	1134	1130	1.30	0.52	0.08	0.19	0.05	0.25	0.00	0.01	0.07	0.08	0.08	0.13	0.07	0.09	0.02	0.06
1,3,8-*p*-Menthatriene	1116	1119	0.52	0.27	4.65	2.02	0.05	0.01	0.17	0.75	0.03	0.22	0.25	1.10	0.12	0.43	0	0.05
(*E*)-2-Nonenal	1130	1134	0.88	0.50	2.61	2.26	0.52	0.04	0.62	0.08	0.19	0.25	0.11	0.55	0.21	0.04	5.67	0.81
*δ*-Terpineol	1145	1148	0.20	0.03	0.33	0.17	0.19	0.07	0.50	0.07	0.36	0.27	0.17	0.34	0.10	0.07	0.14	0.13
Neomentol	1154	1156	0.12	0.26	0.24	0.41	0.07	0.23	0.02	0.03	0.12	0.04	0.02	0.32	0.05	0.02	0.03	0.01
Methyl Salicylate	1173	1171	14.49	24.31	0.95	4.08	36.62	0.00	1.25	19.05	20.57	0.24	0.17	2.73	0.29	2.26	0.21	72.83
*α*-Terpineol	1175	1176	0.02	0.13	0.39	2.01	0.16	0.77	0.10	0.07	0.47	0.64	0.02	3.57	0.50	0.01	0.15	0.18
*β*-Cyclocitral	1196	1194	1.67	1.82	0.63	1.01	0.66	0.11	0.36	0.53	0.60	0.81	0.22	1.02	0.50	0.31	0.53	0.16
Nerol	1212	1210	0.15	0.51	1.85	3.04	2.36	0.00	2.55	0.29	0.21	3.70	2.25	1.81	1.75	0.07	4.79	0.57
Thymoquinone	1216	1215	2.19	0.39	6.50	13.74	9.16	0.79	1.41	0.93	0.11	0.39	1.23	0.73	25.64	0.46	2.13	3.83
Cumin aldehyde	1219	1217	0.48	0.73	0.70	2.32	0.28	0.02	0.16	0.16	0.47	0.38	0.16	0.65	0.10	0.01	0.20	0.29
*β*-Homocyclocitral	1234	1236	0.07	0.32	0.07	0.15	0.07	0.01	0.03	0.08	0.11	0.48	0.03	0.23	0	0.07	0.05	0.02
(*E*)-2-Decenal,	1238	1240	1.60	4.66	4.98	4.50	1.75	0.05	5.65	2.39	0.61	3.47	3.27	2.71	1.61	0.18	27.95	2.49
2-Hydroxy-benzoic acid ethyl ester	1251	1252	1.42	0.61	0.97	0.19	9.57	0.01	0.93	1.64	0.13	0.21	0.90	0.74	0.34	0.06	1.24	0.30
*p*-Ethyl-benzyl alcohol, -	1259	n.d.	0.05	0.04	0.01	0.04	0.01	0.13	0.00	0.01	0.02	0.03	0.07	0.00	0.02	0.01	0.02	0.05
2-Undecanone	1270	1273	0.99	1.67	0.23	0.54	0.24	0.02	0.00	0.02	0.15	0.08	0.03	0.09	0.08	0.07	0.22	0.24
Terpinene 4-acetate	1291	1290	0.02	0.07	0.00	0.16	0.01	0.01	0.05	0.01	0.28	0.11	0.36	0.38	0.41	0.17	0.04	0.28
2-Undecanol	1300	1301	2.67	2.26	2.35	4.64	1.88	0.09	1.88	0.75	0.33	0.99	0.97	1.24	1.32	0.53	3.74	0.93
3-Undecanol	1305	1308	0.12	0.14	0.52	0.07	0.04	0.01	0.05	0.13	0.03	0.07	0.06	0.16	0.05	0.02	0.11	0.04
1-Phenyl-1-pentanone,	1325	1327	0.46	1.53	0.06	0.59	0.14	0.01	0.00	0.02	0.20	0.58	0.04	0.22	0.06	0.12	0.09	0.10
* (*E*)-2-undecenal,	1355	1357	0.36	1.01	0.18	0.09	0.07	0.04	0	0.08	0.08	0.18	0.01	0.11	0.11	0.02	0.25	0.15
Copaene	1372	1375	0.00	0.36	1.44	0.01	0.12	0.01	0.06	1.46	0.07	0.04	0.02	0.55	0	0.11	0.01	0.06
Isolongifolene	1396	1394	0.38	0.01	2.14	2.62	0.70	0.04	0.33	0.03	0.08	0.02	0.03	2.56	6.73	0.02	0.24	0.94
*α*-Ionone	1432	1411	3.00	0.85	0.02	0.08	0.04	0.13	0.05	0.47	0.35	0.11	0.18	0.73	0.01	0.36	0.07	0.06
*β*-Copaene	1426	1426	0.00	0.01	0.02	0.04	0.03	0.01	0.13	0.00	0.09	0.00	0.00	0.01	0.02	0.02	0.01	0.03
Dihydropseudoionone	1428	1432	1.51	2.69	1.13	3.62	0.56	0.39	0.56	0.40	0.25	0.86	0.24	0.99	0.33	0.10	1.73	0.72
*β*-ionone	1461	1466	1.78	2.65	1.41	1.60	0.51	0.13	0.61	1.11	0.53	1.10	0.44	2.50	0.94	0.49	0.44	0.15
*γ*-Guaiene	1498	1499	0.72	0.36	0.16	0.47	0.22	0.04	0.21	0.70	0.27	0.07	0.17	0.01	0.07	0.22	0.36	0.33
*δ*-Cadinene	1516	1517	0.14	0.07	0.19	0.23	0.10	0.15	0.00	0.73	0.04	0.01	0.03	0.10	0.31	0.06	0.04	0.00
**Sum of components group**																		
**non-terpene aliphatic alcohols**			8.45	4.73	26.45	7.14	5.68	7.57	23.24	21.95	30.43	18.58	36.57	28.55	26.4	21.12	18.35	2.01
**non-terpene aliphatic aldehydes and ketones**			35.79	35.7	21.58	21.1	21.14	76.39	12.23	34.99	7.61	29.51	46.12	26.24	41.18	57.03	46.29	7.42
**non-terpene aliphatic** **components**			44.24	40.43	48.03	28.24	26.82	83.96	35.47	56.94	38.04	48.09	82.69	54.79	67.58	78.15	64.64	9.43
**Aromatic compounds**			25.23	40.45	17.63	27.09	52.96	10.67	14.3	32.61	51.51	35.37	3.56	22.8	18.93	16.5	18.89	79.35
**Terpenes and terpenoids**			30.05	17.67	34.28	44.09	20.1	5.38	50.16	10.38	10.24	15.96	13.74	22.15	13.45	5.23	16.4	11.13

**Samples abbreviation:** I.aq.F—*Ilex aquifolium*, female specimen; I × m.F—*I.* × *meservae*, female specimen; I × m.M—*I*. × *meservae*, male specimen; I × alt.F—*I*. × *altaclarensis*, female specimen; I.pe.M—*I*. *perneyi*, male specimen. **Shorts:** LRI—literature retention index; ERI—experimental retention index; *—component with unclear identification.

**Table 2:** Plant Taxa used in the study.

Abbreviation	Sex	*Ilex* Species	Photo
**I.aq.F1**	***F***	***Ilex aquifolium* ‘Alaska’** female variety, very valuable due to the glossy leaves, slow growth and high resistance to low temperatures up to −29 degrees	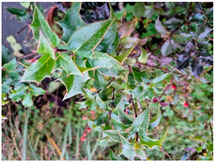
**I × m.M2**	***M***	***Ilex × meserveae*** seedling growing in the collection, male individual grown from seeds collected from variety **‘Blue Girl’**.	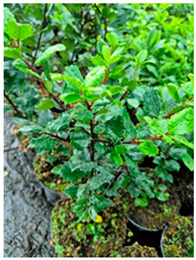
**I × m.F2**	***F***	***Ilex × meserveae* ‘Blue Girl’** mother plant growing in the collection, forming red fruits, dense shrub with slow growth	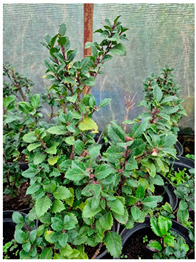
**I × m.F3**	***F***	***Ilex × meserveae* ‘Blue Girl’** seeding growing in collection, female shrub	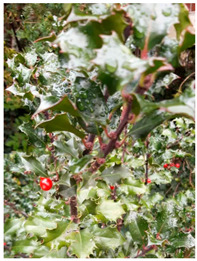
**I.aq.F2**	***F***	***Ilex aquifolium*** female seedling growing in the collection	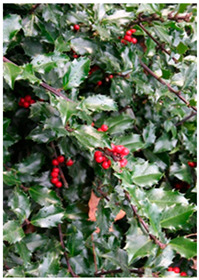
**I × m.F4**	***F***	***Ilex × meserveae* ‘Golden Girl’** female seedling growing in the foil tunnel, cultivated for collecting shoots for cuttings	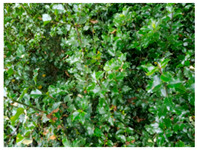
**I × m.F5**	***F***	***Ilex × meserveae* ‘Blue Girl’** female variety with a dense growth, forming fruits, cultivated in unheated foil tunnel for collecting shoots for cuttings	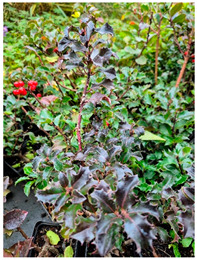
**I × m.F1**	***F***	***Ilex × meserveae* ‘Blue Angel’** female variety with red fruits, slow growth, cultivated in unheated foil tunnel for collecting shoots for cuttings	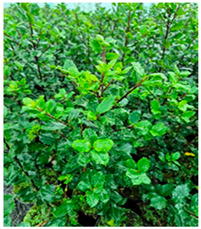
**I.aq.F6**	***F***	***Ilex aquifolium* ‘Pyramidalis Aurea Marginata’** variety with yellow-colored leaves margins with dense, pyramidal growth, cultivated in unheated foil tunnel for collecting shoots for cuttings	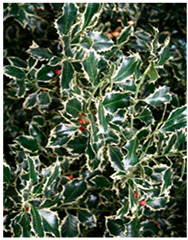
**I.aq.F3**	***F***	***Ilex aquifolium* ‘Aurea Marginata’** shrubs with yellow-colored leaves margins, red fruits, female individual	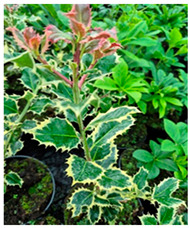
**I × alt.F**	***F***	***Ilex × altaclarensis* ‘Lawsoniana’** a hybrid between *Ilex aquifolium* and *Ilex perado*, occurring in Madeira, Canary Island and Azores. Female variety cultivated in unheated foil tunnel due to better wintering of shrubs, which shoots can freeze during cold winters	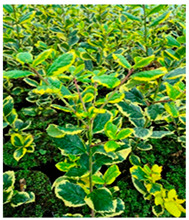
**I.pe.M**	***M***	***Ilex perneyi* Franch** coming from Mongolia and Northeast China, where it forms high shrubs or low trees up to 7 m. In Poland is not frost-resistant, during cold winters shrubs freeze to the snow surface level	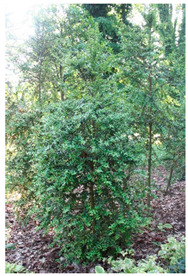
**I.aq.F4**	***F***	***Ilex aquifolium*** female seedling cultivated in foil tunnel	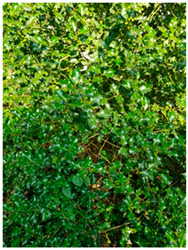
**I.aq.F5**	***F***	***Ilex aquifolium* ‘Alaska’** shrub cultivated in unheated foil tunnel	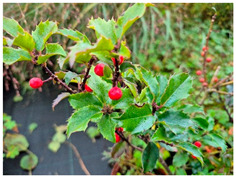
**I × m.F6**	***F***	***Ilex × meserveae* ‘Mesgolg’** shrubs with hard, broadly elliptical leaves length 2–4 cm, sharp top and cuneatic base, with serrated margins (6–8 thorns), nearly flat leaf blade. Dark green top of the blade, bottom light green with blue tone. Variety with female flowers, blooming in the second half of May and on the beginning of June, forms lemon yellow fruits	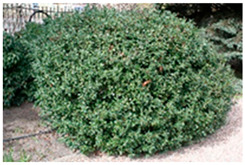
**I × m.M1**	***M***	***Ilex × meserveae* ‘Mesan’** male variety with evergreen leaves, hard, oval or broadly elliptical, with serrated margins (6–9 thorns), glossy top and light green bottom of leaf blade	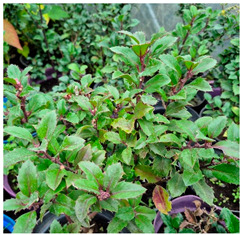

## Data Availability

Research data is available from authors.

## Supplementary Materials

The following supporting information can be downloaded at: https://www.mdpi.com/article/10.3390/molecules30214230/s1, Table S1: PCA 3 factor model; Table S2: Correlation matrix; Table S3: Table with compounds; Table S4: Chromatograms.

## References

[1] Yao X., Song Y., Yang J.B., Tan Y.H., Corlett R.T. (2021). Phylogeny and biogeography of the hollies (*Ilex* L., Aquifoliaceae). J. Syst. Evol..

[2] Yi F., Zhao X., Peng Y., Xiao P. (2016). *Genus llex* L.: Phytochemistry, Ethnopharmacology, and Pharmacology. Chin. Herbal. Med..

[3] Purcaro G., Tranchida P.Q., Jacques R.A., Caramão E.B., Moret S., Conte L., Dugo P., Dugo G., Mondello L. (2009). Characterization of the yerba mate (*Ilex paraguariensis*) volatile fraction using solid-phase microextraction-comprehensive 2-D GC-MS. J. Sep. Sci..

[4] Wang D., Liu C., Li J.J., Guo D.Q., Du H.H. (2021). Complete chloroplast genome sequence and phylogenetic analysis of *Ilex pernyi* Franch. Mitochondrial DNA B Resour..

[5] Salejda A.M., Szmaja A., Bobak Ł., Zwyrzykowska-Wodzińska A., Fudali A., Bąbelewski P., Bienkiewicz M., Krasnowska G. (2021). Effect of *Ilex* × *meserveae* aqueous extract on the quality of dry-aged beef. J. Food Qual..

[6] Márquez V., Martínez N., Guerra M., Fariña L., Boido E., Dellacassa E. (2013). Characterization of aroma-impact compounds in yerba mate (*Ilex paraguariensis*) using GC-olfactometry and GC-MS. Food Res. Int..

[7] Kawakami M., Kobayashi A. (1991). Volatile Constituents of Green Mate and Roasted Mate. J. Agric. Food Chem..

[8] Wei J.F., Gu H.P., Kang W.Y. (2013). Analysis of volatiles in the male flower of *Ilex cornuta* by HS-SPME-GC-MS. Chem. Nat. Compd..

[9] Araújo H.C., Lacerda M.E.G., Lopes D., Bizzo H.R., Kaplan M.A. (2007). Studies on the aroma of maté (*Ilex paraguariensis* St. Hil.) using headspace solid-phase microextraction. Phytochem. Anal..

[10] Galle F.C. (1997). Hollies. The Genus Ilex.

[11] Zwyrzykowska-Wodzińska A., Jarosz B., Okińczyc P., Szperlik J., Bąbelewski P., Zadák Z., Jankowska-Mąkosa A., Knecht D. (2024). GC-MS and PCA Analysis of Fatty Acid Profile in Various *Ilex* Species. Molecules.

[12] Rowan D.D. (2011). Volatile Metabolites. Metabolites.

[13] Pachura N., Kupczyński R., Sycz J., Kuklińska A., Zwyrzykowska-Wodzińska A., Wińska K., Owczarek A., Kuropka P., Nowaczyk R., Bąbelewski P. (2022). Biological Potential and Chemical Profile of European Varieties of *Ilex*. Foods.

[14] Paluch E., Okińczyc P., Zwyrzykowska-Wodzińska A., Szperlik J., Żarowska B., Duda-Madej A., Bąbelewski P., Włodarczyk M., Wojtasik W., Kupczyński R. (2021). Composition and Antimicrobial Activity of *Ilex* Leaves Water Extracts. Molecules.

[15] Kuropka P., Zwyrzykowska-Wodzińska A., Kupczyński R., Włodarczyk M., Szumny A., Nowaczyk R.M. (2021). The Effect of *Ilex × meserveae* S. Y. Hu Extract and Its Fractions on Renal Morphology in Rats Fed with Normal and High-Cholesterol Diet. Foods.

[16] Zwyrzykowska A., Kupczyński R., Jarosz B., Szumny A., Kucharska A.Z. (2015). Qualitative and quantitative analysis of polyphenolic compounds in Ilex sp.. Open Chem..

[17] Hao X., Wang S., Fu Y., Liu Y., Shen H., Jiang L., McLamore E.S., Shen Y. (2024). The WRKY46-MYC2 module plays a critical role in E-2-hexenal-induced anti-herbivore responses by promoting flavonoid accumulation. Plant Commun..

[18] Patrignani F., Iucci L., Belletti N., Gardini F., Guerzoni M.E., Lanciotti R. (2008). Effects of sub-lethal concentrations of hexanal and 2-(*E*)-hexenal on membrane fatty acid composition and volatile compounds of *Listeria monocytogenes*, *Staphylococcus aureus*, *Salmonella enteritidis* and *Escherichia coli*. Int. J. Food Microbiol..

[19] Lanciotti R., Gianotti A., Patrignani F., Belletti N., Guerzoni M.E., Gardini F. (2004). Use of natural aroma compounds to improve shelf-life and safety of minimally processed fruits. Trends Food Sci. Technol..

[20] Spyropoulou E.A., Dekker H.L., Steemers L., van Maarseveen J.H., de Koster C.G., Haring M.A., Schuurink R.C., Allmann S. (2017). Identification and Characterization of (3Z):(2E)-Hexenal Isomerases from Cucumber. Front. Plant Sci..

[21] Qu X., Wang X., Fu M., Cheng J., Liu J., Wang X., Li J., Wang J., Wang Z., Sun F. (2023). (*E*)-2-hexenal regulates the chloroplast degradation in tomatoes. Sci. Hortic..

[22] Gondor O.K., Pál M., Janda T., Szalai G. (2022). The role of methyl salicylate in plant growth under stress conditions. J. Plant Physiol..

[23] Liu P.P., Yang Y., Pichersky E., Klessig D.F. (2010). Altering expression of benzoic acid/salicylic acid carboxyl methyltransferase 1 compromises systemic acquired resistance and PAMP-triggered immunity in *Arabidopsis*. Mol. Plant-Microbe Interact..

[24] Singewar K., Fladung M., Robischon M. (2021). Methyl salicylate as a signaling compound that contributes to forest ecosystem stability. Trees.

[25] Llusia J., Penuelas J., Munné-Bosch S. (2005). Sustained accumulation of methyl salicylate alters antioxidant protection and reduces tolerance of holm oak to heat stress. Physiol. Plant..

[26] Ament K., Krasikov V., Allmann S., Rep M., Takken F.L., Schuurink R.C. (2010). Methyl salicylate production in tomato affects biotic interactions. Plant J..

[27] Gao Z., Bai L., Xu X., Feng B., Cao R., Zhao W., Zhang J., Xing W., Yang X. (2025). The diverse enzymatic targets of the essential oils of *Ilex purpurea* and Cymbopogon martini and the major components potentially mitigated the resistance development in tick Haemaphysalis longicornis. Pestic. Biochem. Physiol..

[28] Gao Z., Yu Z., Qiao Y., Bai L., Song X., Shi Y., Li X., Pang B., Ayiguli M., Yang X. (2022). Chemical profiles and enzyme-targeting acaricidal properties of essential oils from Syzygium aromaticum, *Ilex chinensis* and Citrus limon against Haemaphysalis longicornis (Acari: Ixodidae). Ind. Crops Prod..

[29] Xin Z., Li X., Li J., Chen Z., Sun X. (2016). Application of chemical elicitor (*Z*)-3-hexenol enhances direct and indirect plant defenses against tea geometrid Ectropis obliqua. BioControl.

[30] Xin Z., Ge L., Chen S., Sun X. (2019). Enhanced transcriptome responses in herbivore-infested tea plants by the green leaf volatile (*Z*)-3-hexenol. J. Plant Res..

[31] Liao Y., Tan H., Jian G., Zhou X., Huo L., Jia Y., Zeng L., Yang Z. (2021). Herbivore-Induced (*Z*)-3-Hexen-1-ol is an Airborne Signal That Promotes Direct and Indirect Defenses in Tea (*Camellia sinensis*) under Light. J. Agri. Food Chem..

[32] Jing T., Zhang N., Gao T., Zhao M., Jin J., Chen Y., Xu M., Wan X., Schwab W., Song C. (2019). Glucosylation of (*Z*)-3-hexenol informs intraspecies interactions in plants: A case study in *Camellia sinensis*. Plant Cell Environ..

[33] Ruther J., Kleier S. (2005). Plant–plant signaling: Ethylene synergizes volatile emission in Zea mays induced by exposure to (*Z*)-3-hexen-1-ol. J. Chem. Ecol..

[34] Cofer T.M., Tumlinson J.H. (2025). The carboxylesterase AtCXE12 converts volatile (*Z*)-3-hexenyl acetate to (*Z*)-3-hexenol in *Arabidopsis* leaves. Plant Physiol..

[35] Scalliet G., Lionnet C., Le Bechec M., Dutron L., Magnard J.L., Baudino S., Bergougnoux V., Jullien F., Chambrier P., Vergne P. (2006). Role of petal-specific orcinol O-methyltransferases in the evolution of rose scent. Plant Physiol..

[36] Gupta A.K., Akhtar T.A., Widmer A., Pichersky E., Schiestl F.P. (2012). Identification of white campion (*Silene latifolia*) guaiacol O-methyltransferase involved in the biosynthesis of veratrole, a key volatile for pollinator attraction. BMC Plant Biol..

[37] Mao K., Li C., Zhai H., Wang Y., Lou Y., Xue W., Zhou G. (2024). OsRCI-1-Mediated GLVs enhance rice resistance to brown planthoppers. Plants.

[38] Sun L., Gu S.H., Xiao H.J., Zhou J.J., Guo Y.Y., Liu Z.W., Zhang Y.J. (2013). The preferential binding of a sensory organ specific odorant binding protein of the alfalfa plant bug Adelphocoris lineolatus AlinOBP10 to biologically active host plant volatiles. J. Chem. Ecol..

[39] Jain A., Dhruw L., Sinha P., Pradhan A., Sharma R., Gupta B. (2021). Nutraceuticals.

[40] Saleh A.Y., Sultan S.J., Al-Najim A.N., Sultan S.M., Sultan N.M., Haddad M.F., Saadi A.M. (2025). Synergistic Antifungal Activity of Thymoquinone and Infrared Radiation Against *Aspergillus flavus*. Int. J. Des. Nat. Ecodyn..

[41] Kumar A.R., Martínez-López W., Saraswathy R. (2023). Biotechnology for Toxicity Remediation and Environmental Sustainability.

[42] Ashour H., Zafrah H., Dafaalla Mohammed M.E., Rashed L.A., Abbas A.M., Kamar S.S., ShamsEldeen A.M. (2025). Ferrostatin-1 Partially Suppressed the Anti-Fibrotic Actions of Thymoquinone in a Rat Model of Cholestasis-Induced Liver Injury. Int. J. Morphol..

[43] Sun C., Chen J., Wang L., Li J., Shi Z., Yang L., Yu X. (2024). Thymol deploys multiple antioxidative systems to suppress ROS accumulation in Chinese cabbage seedlings under saline stress. Agronomy.

[44] Butnariu M., Quispe C., Herrera-Bravo J., Helon P., Kukula-Koch W., López V., Les F., Vergara C.V., Alarcón-Zapata P., Alarcón-Zapata B. (2022). The effects of thymoquinone on pancreatic cancer: Evidence from preclinical studies. Biomed. Pharmacother..

[45] Barkat M.A., Harshita, Pottoo F.H., Beg S., Rahman M., Ahmad F.J. (2020). Nanomedicine for Bioactives: Healthcare Applications.

[46] Bimonte S., Albino V., Barbieri A., Tamma M.L., Nasto A., Palaia R., Molino C., Bianco P., Vitale A., Schiano R. (2019). Dissecting the roles of thymoquinone on the prevention and the treatment of hepatocellular carcinoma: An overview on the current state of knowledge. Infect. Agents Cancer.

[47] Sharma B., Shekhar H., Sahu A., Haque S., Kaur D., Tuli H.S., Sharma U. (2025). Deciphering the anticancer potential of thymoquinone: In-depth exploration of the potent flavonoid from *Nigella sativa*. Mol. Biol. Rep..

[48] Yi T., Cho S.G., Yi Z., Pang X., Rodriguez M., Wang Y., Sethi G., Aggarwal B.B., Liu M. (2008). Thymoquinone inhibits tumor angiogenesis and tumor growth through suppressing AKT and extracellular signal-regulated kinase signaling pathways. Mol. Cancer Ther..

[49] Rahmani A.H. (2018). Molecular and Therapeutic actions of Thymoquinone: Actions of Thymoquinone.

[50] Almatroodi S.A., Almatroudi A., Alsahli M.A., Khan A.A., Rahmani A.H. (2020). Thymoquinone, an active compound of Nigella sativa: Role in prevention and treatment of cancer. Curr. Pharm. Biotechnol..

[51] Sundaravadivelu S., Raj S.K., Kumar B.S., Arumugamand P., Ragunathan P.P. (2019). Reverse screening bioinformatics approach to identify potential anti breast cancer targets using thymoquinone from neutraceuticals Black Cumin Oil. Anti-Cancer Agents Med. Chem..

[52] Tariq H., Chen P., Wang G., Xie S., Wang L., Lv J., Wang F., Bilal M.S., Khan A.R., Rajput N.A. (2025). Trans-2-decenal inhibits *Alternaria alternata* through disruption of redox homeostasis and membrane integrity. Pest Manag. Sci..

[53] Slavković F., Bendahmane A. (2023). Floral phytochemistry: Impact of volatile organic compounds and nectar secondary metabolites on pollinator behavior and health. Chem. Biodivers..

[54] Al-Khalili M., Pathare P.B., Rahman S., Al-Habsi N. (2025). Aroma compounds in food: Analysis, characterization and flavor perception. Meas. Food.

[55] Gerber T., Nunes A., Moreira B.R., Maraschin M. (2023). Yerba mate (*Ilex paraguariensis* A. St.-Hil.) for new therapeutic and nutraceutical interventions: A review of patents issued in the last 20 years (2000–2020). Phytother. Res..

